# Detection of circulating tumor cells in colorectal cancer patients using the GILUPI CellCollector: results from a prospective, single‐center study

**DOI:** 10.1002/1878-0261.12507

**Published:** 2019-06-17

**Authors:** Levent Dizdar, Georg Fluegen, Guus van Dalum, Ellen Honisch, Rui P. Neves, Dieter Niederacher, Hans Neubauer, Tanja Fehm, Alexander Rehders, Andreas Krieg, Wolfram T. Knoefel, Nikolas H. Stoecklein

**Affiliations:** ^1^ Department of General, Visceral and Pediatric Surgery University Hospital and Medical Faculty of the Heinrich‐Heine‐University Düsseldorf Germany; ^2^ Department of Obstetrics and Gynecology University Hospital and Medical Faculty of the Heinrich‐Heine‐University Düsseldorf Germany

**Keywords:** CellCollector, CellSearch, colorectal cancer, CTC, prospective study

## Abstract

The GILUPI CellCollector (CC) is a novel *in vivo* circulating tumor cell (CTC) detection device reported to overcome the limitations of small blood sample volumes. The aim of this prospective, blinded study was to evaluate the clinical application of the CC and to compare its performance to the CellSearch (CS) system in M0 and M1 colorectal cancer (CRC) patients. A total of 80 patients (31 M0, 49 M1) with CRC were enrolled. CTCs were simultaneously measured in the peripheral blood using CS and the CC, and the results of both assays were correlated to clinicopathological variables and overall survival. The total number of detected CTCs and CTC‐positive patients did not significantly differ between both assays. In the M0 patients, the CC detected CTCs more frequently than CS. There was no significant difference in total CTC numbers detected with the CC between M0 and M1 patients. In addition, no significant correlation with clinicopathological parameters or overall survival was observed with CC CTCs. In contrast, detection of CTCs with CS was significantly correlated with Union for International Cancer Control stage and reduced overall survival. There was no correlation between CTCs detected by the CC and the CS system. Using *in silico* analysis, we estimate that CC screens a volume of 0.33–18 mL during *in vivo* application, in contrast to much higher volumes reported elsewhere. In conclusion, while being safe and easy to use, the CC did not outperform CS in terms of CTC yield or sensitivity. While CTC detection in M0 CRC patients was significantly increased with the CC, the clinical relevance of these CTCs appears inferior to the cells identified by the CS system.

**Abbreviations**
CC: CellCollector^®^
CD45: lymphocyte common antigen
CI: confidence interval
CRC: colorectal cancer
CS: CellSearch®
CTC: circulating tumor cell
DAPI: 4,6‐diamidino‐2‐phenylindole
DLA: diagnostic leukapheresis
EpCAM: epithelial cell adhesion molecule
FDA: U.S. Food and Drug Administration
HR: hazard ratio
*n*: number
panCK: pan‐cytokeratin
PCR: polymerase chain reaction
SEM: standard error of the mean
UICC: Union for International Cancer Control
yrs: years

## 1 Introduction

Circulating tumor cells (CTC) enumerated by the CellSearch (CS) system have significant prognostic impact in colorectal cancer (CRC) and are considered promising biomarkers for the management of this disease. In localized CRC, the detection of CTCs could help to identify those patients at risk for metastasis and to stratify them to adjuvant therapies. In advanced metastatic CRC, the enumeration of CTCs can improve risk assessment, monitoring of systemic therapy, and detection of therapy resistance (Bork *et al*., 2015; Cristofanilli *et al*., 2004; Danila *et al*., 2007; Gazzaniga *et al*., 2013; Maheswaran *et al*., 2008; Tsai *et al*., 2016). Beyond enumeration, a far greater potential for CTC‐based liquid biopsies lies in subsequent molecular characterization of detected CTCs, for example, assessing resistance‐conferring mutations for anti‐epidermal growth factor receptor therapies (Bork *et al*., 2015; Cohen *et al*., 2009; Gazzaniga *et al*., 2013; Gorges *et al*., 2016; Hall *et al*., 2016; Krebs *et al*., 2015; Lucci *et al*., 2012; Romiti *et al*., 2014; Scher *et al*., 2015; Scherag *et al*., 2017; Tsai *et al*., 2016). However, an important unresolved challenge limiting the widespread use of CTC‐based liquid biopsies in clinical routine is their infrequent and unreliable detection, which has been mainly attributed to the low volume of the investigated blood samples (Stoecklein *et al*., 2016).

One approach to sample high volumes of blood without negative consequences for the patient is the diagnostic leukapheresis (DLA) (Fischer *et al*., 2013). Yet, relatively high costs, a required specialized infrastructure, and the need for trained personnel may restrict the use of DLA to dedicated centers. In this context, the GILUPI CellCollector (CC), a functionalized medical wire covered with anti‐ epithelial cell adhesion molecule (EpCAM) antibodies, appears as a simpler method to increase the analyzed blood volume (Gorges *et al*., 2016; Saucedo‐Zeni *et al*., 2012). This *in vivo* wire is designed to capture CTCs directly from the peripheral blood of cancer patients while being inserted into the cubital vein for 30 min. So far, *ex vivo* spiking experiments (Scherag *et al*., 2017) and *in vivo* studies have reported promising detection rates and higher CTC yields when compared to standard methods (Gorges *et al*., 2016; Kuske *et al*., 2016; Mandair *et al*., 2016; Scherag *et al*., 2017), attributed to higher blood volumes screened *in vivo* (1–3 L) (Gorges *et al*., 2016; Kuske *et al*., 2016; Saucedo‐Zeni *et al*., 2012). However, the relevance of CC‐detected CTCs in CRC remains unclear. Therefore, we conducted this prospective, investigator‐blinded study in an unselected cohort of CRC patients to analyze the detection frequency and prognostic impact of CC CTCs and to compare these side by side with the FDA‐cleared CS system. In analogy to previous reports and in accordance with the reported high blood volume screened, we anticipated a higher CTC detection rate in the CC arm of the study, especially in the nonmetastatic CRC group.

## 2 Materials and methods

### 2.1 Study design

This prospective, investigator‐blinded, single‐center clinical study conducted at the Department of General, Visceral and Pediatric Surgery of the University Hospital Düsseldorf, Germany, investigated CTC detection in CRC patients using the CC, a novel *in vivo* device for CTC detection, and the FDA‐approved CS system. The results of both CTC assays were compared and correlated to clinicopathological data as well as patients’ overall survival. The study was carried out in accordance with Good Clinical Practice guidelines and the Declaration of Helsinki and was approved by the Ethics Committee of the Medical Faculty of the Heinrich‐Heine‐University Düsseldorf (Ref‐No: 3958/2013). All patients included provided written informed consent.

### 2.2 Patients

A total of 80 (49 male and 31 female) consecutive patients with histologically confirmed CRC were included in this study between February 2013 and June 2016. Patients were in varying stages of their treatment at the department of surgery. Patients with a history of another malignancy within the last 5 years were excluded. CRCs were staged according to the 7th edition of the Union for International Cancer Control (UICC) TNM classification of malignant tumors (Sobin *et al*., 2010) by experienced pathologists. The median age of all patients was 70 years (range 36–91 years). Sixty‐one patients presented with primary tumors. Of these, 29 patients were diagnosed with nonmetastatic disease (UICC stage I–III) and 32 patients with synchronous distant metastases (UICC stage IV). Furthermore, two patients with locally recurrent rectal cancer and 17 patients with metastatic cancer relapses were included. Complete oncological resection of the tumor (R0) was achieved in 60 patients. Of the remaining 20 patients (R1 + 2), 18 received palliative treatment in late, nonresectable stages of disease, while the other two patients were operated with curative intent, but due to widespread dissemination underwent R1 resection. Of the cohort of 80 CRC patients, 59 patients with R0 resection (median follow‐up of 23.8 months, range 1–48.6 month) and a mean overall survival of 36.6 months (range 1.0–48.6 month; 95% CI: 31.9–41.3) were included into the survival analysis. Of these 59 patients, seventeen patients (28.8%) deceased during the follow‐up period. In addition to the 20 patients with residual tumor masses (R1 + 2), one patient died within the first 30 days after successful R0 surgery and was excluded from the survival analysis. Data regarding overall survival were obtained from a prospectively maintained clinical database curated for this study. Overall survival was defined as the period from the date of CTC detection until death from any cause, or until the date of the last follow‐up.

### 2.3 Blood sample collection and application of the CellCollector

A 20‐G peripheral venous catheter was placed into the cubital vein of the patients, and 5 mL of peripheral blood was drawn and discarded. Subsequently, 7.5 mL of venous blood was collected into a CellSave tube for CS analysis. The CC device was carefully inserted through the catheter until the functionalized tip extended 2 cm into the vein. The wire remained in the cubital vein for 30 minutes, during which the patient stayed in a supine position. After removal from the cubital vein, the device was washed in PBS, fixed with acetone, and stored at −20 °C, according to the manufacturer's instructions. In all patients, blood was collected before surgery together with routine blood samples.

### 2.4 CTC detection

**Figure 1:**
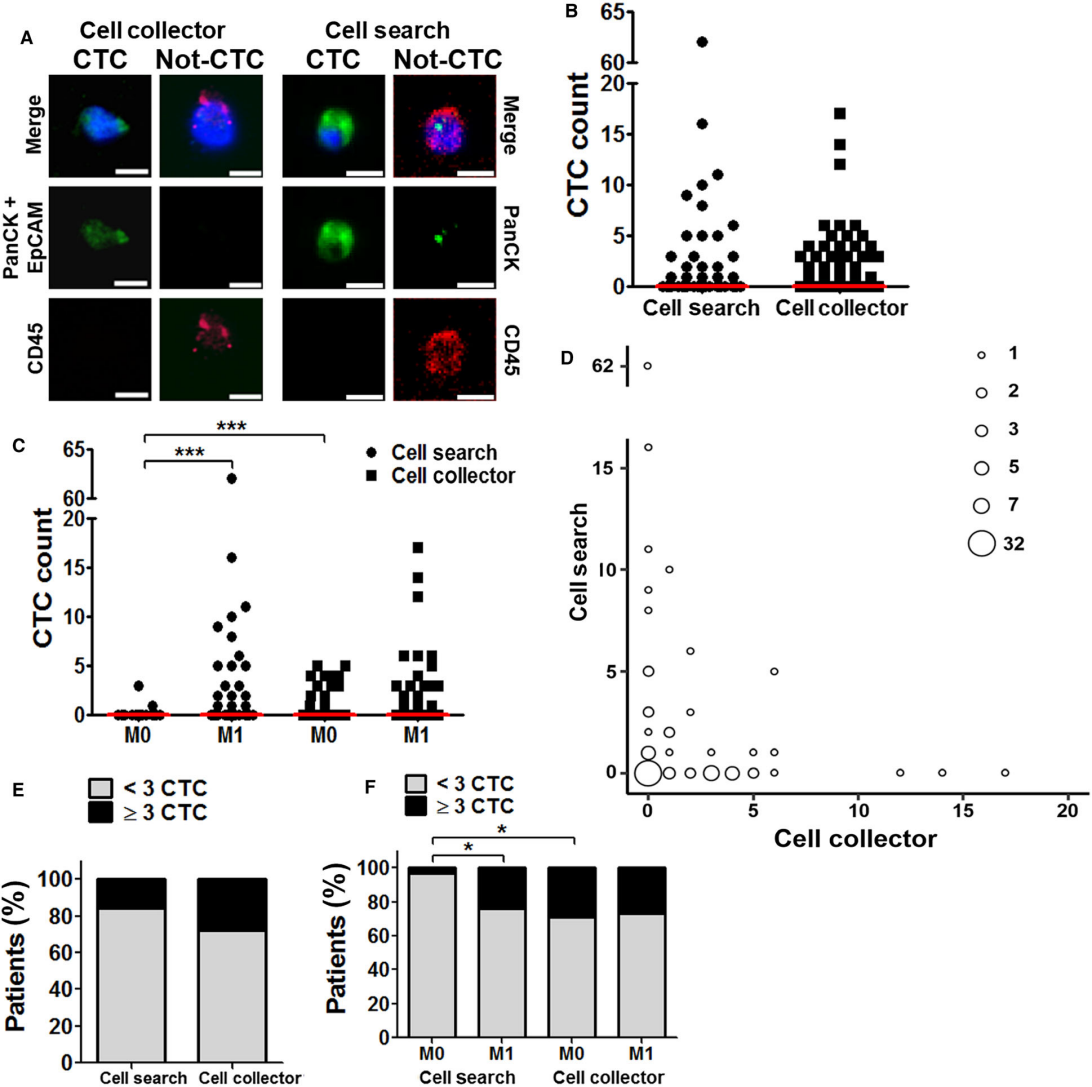
(A) Representative IF images of cells detected with CC and CS (1 CTC, 1 leukocyte). Pan‐CK, CTC marker; EpCAM, CTC marker; CD45, leukocyte marker; merge including Hoechst (CC) or DAPI (CS) nuclear staining. Scale bar 10 μm. (B) Number of CTC per patient detected with CS and CC; *n* = 80, bar shows median. (C) Number of CTC per patient in nonmetastatic (M0) and metastatic (M1) patients detected with CS and CC; *n* = 80, bar shows median. (D) Correlation of CTC number detected per patient using both CS and CC. Each circle represents the number of samples given in the legend. *n* = 80. (E) Bar graph showing percentage of CTC‐positive patients of all included 80 CRC patients. (F) Bar graph showing the percentage of CTC‐positive nonmetastatic (M0) and metastatic (M1) patients for CS and CC. *P* ‐values were calculated by Mann–Whitney test, * *P* < 0.05, *** *P* < 0.001.

For CTC detection using the CS, CellSave tubes were processed by experienced operators within 96 h using the CS CTC Kit, as recommended by the manufacturer. For CTC detection using the CC, applied CCs were sent to GILUPI GmbH, Potsdam, Germany, according to the specifications provided by them, and captured cells were stained and counted by experienced investigators. Importantly, the operators analyzing the CS or CC samples were blinded to the patient's clinicopathological data, as well as to the results obtained by the complementary method. Both methods enrich CTCs based on expression of EpCAM. However, according to respective immunofluorescence‐based protocol for identification of CTCs, CS uses the positivity for pan‐cytokeratin (panCK) staining as a criterion to define CTCs, while CC uses positivity for panCK/EpCAM (double‐staining). Both systems use lymphocyte common antigen (CD45) as marker to exclude hematogenous cells and 4,6‐diamidino‐2‐phenylindole (DAPI)/Hoechst as marker to identify intact cells (Fig. 1A).

We used the established cutoff of ≥ 3 CTCs (Cohen *et al*., 2009) to define CTC‐positive CRC patients in this study. Yet, since only limited data regarding M0 CRC and CTC cutoffs are available, we also calculated the statistical significance using two additional cutoff values (≥ 1 CTC; ≥ 2 CTCs); the results are shown in Table S2.

### 2.5 Statistical analysis

Based on the results of Coumans *et al*. (2012), the prevalence of detecting three or more CTCs with CS in 7.5 mL of blood from metastatic CRC patients is ~ 22–30%. Extrapolating to the reported higher screening volumes of the CC, one can expect a detection frequency of ~ 80–97% for three or more CTCs in 750 mL of blood in metastatic CRC patients. Assuming the CC could screen at least 750 mL of blood *in vivo*, using McNemar's test for categorical variables, we calculated a cohort of 26 metastatic CRC patients to reach a power of 0.95 when comparing the CC results to simultaneous CS samples. Thus, we recruited all consecutive CRC patients irrespective of UICC stage, until 32 metastatic CRC patients were included in the study.

A correlation between CTC detection and clinicopathological variables was examined using the nonparametric Mann–Whitney test for numerical and the chi‐square test for categorical data. Standard error of the mean (SEM) CTC count was used to calculate significance in differences. Differences in CTC detection results obtained by the CC or the CS system were analyzed by Wilcoxon matched pairs test, and Spearman's correlation coefficient was used to test a relationship between the results of both CTC assays.

Kaplan–Meier curves were generated and assessed using the log‐rank (Mantel–Cox) test. For multivariate survival analysis, all variables were included into a forward logistic regression analysis. All variables showing significance in the univariate analysis were included in the multivariate analysis. Results are presented as hazard ratio (HR) with 95% CI and *P*‐values. All statistical analyses were performed using GraphPad Prism for Windows (version 5, graphpad software, San Diego, CA, USA) or spss statistics for Windows (version 17.0, SPSS Inc., Chicago, IL, USA). *P*‐values < 0.05 were considered statistically significant.

## 3 Results

### 3.1 Patients

Patient characteristics are summarized in Table 1. We encountered no adverse effects during the application of the CC or the collection of blood samples for CS, demonstrating that both systems are safe to use within a clinical setting.

**Table 1:** Clinicopathological characteristics of all 80 patients.

Variable	No. of patients (%)
Age (median = 70)	
< 70	38 (47.5)
≥ 70	42 (52.5)
Sex	
Male	49 (61.3)
Female	31 (38.8)
Primary tumor	61 (76.3)
Location	
Colon	37 (60.7)
Rectum	24 (39.3)
Tumor stage	
T1/T2	11 (18.0)
T3/T4	50 (82.0)
Lymph node	
N0	22 (36.1)
N+	39 (63.9)
Distant metastasis	
M0	29 (47.5)
M+	32 (52.5)
UICC stage	
UICC I	8 (13.1)
UICC II	9 (14.8)
UICC III	12 (19.7)
UICC IV	32 (52.5)
Recurrent disease	19 (23.8)
Local recurrence	2 (10.5)
Distant recurrence	17 (89.5)
Treatment intent	
Curative	62 (77.5)
Palliative	18 (22.5)
Resection status	
R0	60 (75.0)
R1	2 (2.5)
R2 (palliative)	18 (22.5)

### 3.2 CTC detection with CC and CS

Representative images of CTCs detected by CS and CC are displayed in Fig. 1A. In total, 161 CTCs were detected by CS and 135 by CC. At least one CTC was detected by CS in 25/80 patients (31.3%) and in 33/80 (41.3%) by CC. There was a significant difference between the two methods neither in the number of CTCs detected per sample nor in the detection frequency (Fig. 1B–E). Despite this, we observed no correlation in the number of CTCs detected with CS and CC in each individual sample using a Spearman's correlation coefficient (Fig. 1D and Table S1).

### 3.3 Calculation of the analyzed blood volume screened by *in vivo* application of CC

Previous publications have suggested that the CC screens a blood volume between one and three liters during the 30‐min *in vivo* exposure (Saucedo‐Zeni *et al*., 2012; Scherag *et al*., 2017), and therefore, we expected a significantly higher CTC detection with the CC. The very similar CTC detection frequencies measured in our study prompted us to estimate the volume of blood in contact with the CC during the 30 min when inserted into the cubital vein. For our 2D and 3D *in silico* approximation (Appendix S1 and Figs S1 and S2), we assumed that the CC device is positioned in the center of the vessel and that it is able to capture any CTC passing within a certain distance of its surface. As this arbitrary assumption is not based on experimental evidence, we calculated the volume for a capture distance of 25, 50, and 75 μm distance. In addition, taking into account the vastly different anatomy of patients as well as the dynamics of the venous vasculature, we considered a range of average blood flow rates in the cubital vein of 4–6 cm·s^−1^ (OpenStaxCollege 2013, Theil *et al*., 2016) in a vessel of 1.7–2.7 mm diameter (OpenStaxCollege 2013). These assumptions resulted in a sampled blood volume in the range between 0.33 and 18 mL for a 30‐min incubation period (Appendix S1), helping to explain the lack of significantly different detection rates between CC and CS.

### 3.4 CTC detection and correlation to clinicopathological parameters

Notably, neither the total number of CTCs (Table 2, Fig. 1B) nor the frequency of positive patients (Table 3, Fig. 1E) detected with the CC correlated significantly with any of the assessed clinicopathological parameters. The number of CTCs detected by CS on the other hand significantly correlated to the relevant clinicopathological parameters assessed in this study. Significantly higher mean CS CTC counts could be observed in patients with lymph node or distant metastasis, higher UICC Stage, and irresectable disease (Table 2). In addition, the frequency of CS CTC‐positive patients in the M1 cohort was significantly higher than in the M0 (Table 3, Fig. 1C,F). Interestingly, in the cohort of M0 patients, the CC detected a significantly higher rate of CTC‐positive patients compared to CS (Table 3, Fig. 1F).

**Table 2:** Mean CTC number and correlating clinicopathological characteristics. *P* ‐values were calculated by Mann–Whitney test for numerical and the chi‐square test for categorical data, SEM, standard error of the mean

	CellSearch		CellCollector	
	Mean CTC count (SEM)	*P* ‐value	Mean CTC count (SEM)	*P* ‐value
Age				
< 70	1.45 (0.47)	0.605	1.47 (0.52)	0.129
≥ 70	2.52 (1.52)		1.88 (0.47)	
Sex				
Male	1.61 (0.48)	0.200	1.76 (0.48)	0.833
Female	2.65 (2.01)		1.58 (0.49)	
Primary tumor				
Location				
Colon	2.97 (1.68)	0.232	1.35 (0.34)	0.913
Rectum	1.54 (0.81)		1.63 (0.64)	
Tumor stage				
T1/T2	0.18 (0.18)	0.051	2.28 (1.27)	0.632
T3/T4	2.90 (1.29)		1.28 (0.28)	
Lymph node				
N0	0.14 (0.10)	**0.001**	1.55 (0.68)	0.701
N+	3.69 (1.64)		1.41 (0.33)	
Distant metastasis				
M0	0.14 (0.11)	**< 0.001**	1.41 (0.34)	0.533
M+	4.47 (1.97)		1.50 (0.54)	
UICC stage				
UICC I/II	0.00 (0.00)	**0.001**	1.12 (0.43)	0.641
UICC III/IV	3.34 (1.46)		1.59 (0.41)	
Recurrent disease				
Local recurrence	0.00 (0.00)	0.455	0.00 (0.00)	0.191
Distant recurrence	0.82 (0.64)		2.71 (1.14)	
Resection status				
R0	0.52 (0.18)	**< 0.001**	1.57 (0.35)	0.837
R1 + 2	6.50 (3.10)		2.05 (0.92)	

Statistical significant values (*P* < 0.05) are shown in bold.

**Table 3:** Correlation of CTC detection with CS or CC and clinicopathological characteristics. *P* ‐values were calculated by Mann–Whitney test for numerical and the chi‐square test for categorical data

Patient subset	CellSearch		CellCollector	
	≥ 3 CTCs	*P* ‐value	≥ 3 CTCs	*P* ‐value
Age (years)	*n* (%)		*n* (%)	
< 70 (*n* = 38)	7 (18.4)	0.617	7 (18.4)	0.084
≥ 70 (*n* = 42)	6 (14.3)		15 (35.7)	
Sex				
Male (*n* = 49)	9 (18.4)	0.519	13 (26.5)	0.807
Female (*n* = 31)	4 (12.9)		9 (29.0)	
Primary tumor				
Location				
Colon (*n* = 37)	9 (24.3)	0.256	9 (24.3)	0.952
Rectum (*n* = 24)	3 (12.5)		6 (25.0)	
Tumor stage				
T1/T2 (*n* = 11)	0 (0.0)	0.070	3 (27.3)	0.819
T3/T4 (*n* = 50)	12 (24.0)		12 (24.0)	
Lymph node				
N0 (*n* = 22)	0 (0.0)	**0.004**	5 (22.7)	0.800
N+ (*n* = 39)	12 (30.8)		10 (25.6)	
Distant metastasis				
M0 (*n* = 29)	1 (3.4)	**0.002**	9 (31.0)	0.266
M+ (*n* = 32)	11 (34.4)		6 (18.8)	
UICC stage				
UICC I/II (*n* = 17)	0 (0.0)	**0.016**	4 (23.5)	0.905
UICC III/IV (*n* = 44)	12 (27.3)		11 (25.0)	
Recurrent disease				
Local recurrence (*n* = 2)	0 (0.0)	0.725	0 (0.0)	0.253
Distant recurrence (*n* = 17)	1 (5.9)		7 (41.2)	
Resection status				
R0 (*n* = 60)	5 (8.3)	**0.001**	17 (28.3)	0.772
R1 + R2 (*n* = 20)	8 (40.0)		5 (25.0)	

Statistical significant values (*P* < 0.05) are shown in bold.

### 3.5 CTC detection and prognostic impact

**Figure 2:**
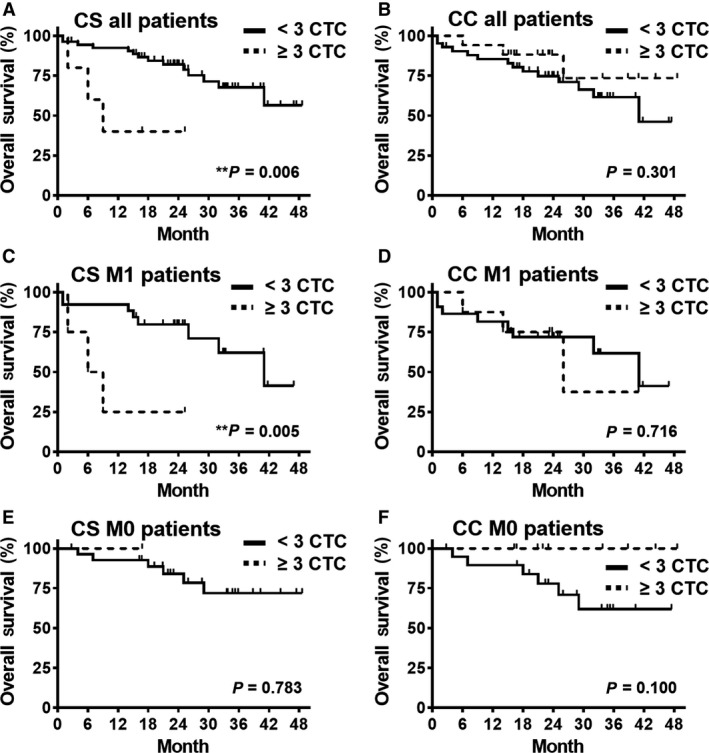
(A) + (B) Kaplan–Meier graph showing overall survival of all 59 primary CRC patients with complete resection (R0) stratified for detection of ≥ 3 CTCs using CS or CC. (C–F) Kaplan–Meier graph showing the same for all 30 metastatic (M1) and all 29 nonmetastatic (M0) patients with R0 resection. One patient with survival < 30 days was excluded. *P* ‐values calculated with log‐rank test and shown in graph.

Univariate survival analysis revealed that the presence of ≥ 3 CTCs detected by the CS system was significantly associated with poor overall survival (Table 4). This finding was confirmed by multivariate logistic regression and Kaplan–Meier analysis (Table 4, Fig. 2A). In contrast, CTCs detected by the CC system were not significantly associated with the overall survival of patients, irrespective of the cutoff (Table 4, Fig. 2B, Table S2). In the subgroup of patients who underwent R0 surgery with curative intent (UICC 1–4, *n* = 59), univariate survival analysis revealed that distant metastasis, but also CTCs detected by CS, was correlated with a significantly reduced survival (Table 4). Upon multivariate logistic regression analysis, the detection of ≥ 3 CS CTCs was of independent prognostic significance (Table 4). In the subgroup of patients with metastatic CRC (M1, *n* = 30), the detection of ≥ 3 CS CTCs was also associated with an unfavorable prognosis in the univariate (Table 4, Fig. 2C) and multivariate (Table 4) analysis, while detection of CTCs with the CC was not correlated to overall survival (Table 4, Fig. 2D). Finally, in the subgroup of patients with nonmetastatic disease (M0, *n* = 29), detection of ≥ 1 CTC by CS correlated significantly with a worse overall survival in the univariate, yet not in the multivariate analysis (Table 4, Fig. 2E). Interestingly, in this same subgroup of patients, detection of ≥ 1 CTC by the CC was even associated with a favorable prognosis in the univariate analysis (Table 4, Fig. 2F).

**Table 4:** Univariate and multivariate survival analysis of all 59 patients with R0 resection. For multivariate survival analysis, age and all clinical variables significant in the univariate analysis were included in a forward logistic regression analysis. In case of more than one CTC cutoff showing significance in the univariate analysis, only the cutoff with the smallest *P* ‐value was used in the multivariate analysis. All variables included in the multivariate analysis are marked with * in the univariate analysis. Results are presented as HR with 95% CI

Variable	Univariate analysis			Multivariate analysis		
	HR	95% CI	*P* value	HR	95% CI	*P* value
All primary tumours (*n* = 59)						
Age*	0.617	0.234–1.630	0.325	/	/	/
Sex	1521	0.558–4.142	0.408			
Distant metastasis	2126	0.784–5.762	0.129			
CellSearch (CTC ≥ 1)	2357	0.865–6.419	0.084			
CellSearch (CTC ≥ 2)	2684	0.871–8.275	0.073			
CellSearch (CTC ≥ 3)*	5141	1.386–19.06	**0.006**	5141	1.386–19.06	**0.014**
CellCollector (CTC ≥ 1)*	0.354	0.115–1.086	0.057	**/**	**/**	**/**
CellCollector (CTC ≥ 2)	0.414	0.119–1.443	0.152			
CellCollector (CTC ≥ 3)	0.524	0.150–1.825	0.301			
Metastatic disease (M+) (*n* = 30)						
Age*	0.768	0.224–2.634	0.673	**/**	**/**	**/**
Sex	0.689	0.205–2.310	0.542			
CellSearch (CTC ≥ 1)	1474	0.440–4.941	0.526			
CellSearch (CTC ≥ 2)	2364	0.680–8.218	0.162			
CellSearch (CTC ≥ 3)*	6342	1.446–27.82	**0.005**	6342	1.446–27.82	**0.014**
CellCollector (CTC ≥ 1)*	0.711	0.207–2.443	0.585	**/**	**/**	**/**
CellCollector (CTC ≥ 2)	1092	0.278–4.283	0.899			
CellCollector (CTC ≥ 3)	1286	0.329–5.031	0.716			
Non‐metastatic disease (M0) (*n* = 29)						
Age*	0.528	0.106–2.627	0.428	**/**	**/**	**/**
Sex	5609	0.654–48.08	0.076			
CellSearch (CTC ≥ 1)*	12 748	0.797–203.8	**0.020**	12 748	0.797–203.8	0.072
CellSearch (CTC ≥ 2)	0.047	0.000–open	0.783			
CellSearch (CTC ≥ 3)	0.047	0.000–open	0.783			
CellCollector (CTC ≥ 1)*	0.019	0.000–14.42	**0.029**	**/**	**/**	**/**
CellCollector (CTC ≥ 2)	0.023	0.000–22.39	0.057			
CellCollector (CTC ≥ 3)	0.027	0.000–39.91	0.100			

Statistical significant values (*P* < 0.05) are shown in bold.

## 4 Discussion

The previously reported lower number and frequency of CTCs detected with FDA‐cleared CS in patients with metastatic CRC limit the informative potential of CTCs in this disease. In part, the low detection is a consequence of the low volume of blood sampled for the standard analysis (van Dalum *et al*., 2015; Stoecklein *et al*., 2016). We have previously reported the value of DLA to increase the number of CTCs detected in patients due to the analysis of a larger volume of blood (Fischer *et al*., 2013). Aiming at a logistically much simpler alternative to sample large volumes of blood that ultimately could be used as point‐of‐care CTC test, we have evaluated the GILUPI CC. This system has been previously suggested to interrogate one to three liters of blood and was shown to increase both the number and detection rate of CTCs in lung, breast, and prostate cancer (Gorges *et al*., 2016; Kuske *et al*., 2016; Saucedo‐Zeni *et al*., 2012). In this context, very surprisingly, in our prospective and investigator‐blinded side‐by‐side comparison of CC with CS in metastatic and nonmetastatic CRC patients, we could find a significant difference neither in total number, nor in the frequency of CTCs detected by both methods. The observed lack of increase in CTC detection prompted us to estimate the blood volume in contact with the CC during the 30‐min *in vivo* application in the cubital vein. We used the technical information supplied by the CC manufacturer as well as vascular parameters previously published to mathematically model the volume sampled. Our estimates based on two simplified models indicated a screened blood volume between 0.33 and 18 mL (Appendix S1). Importantly, CTC capture efficiency, interpatient variability in blood flow and vein diameter caused by the position of the wire (Nifong and McDevitt, 2011), or interpatient differences in blood viscosity were not modeled. Such variables will additionally impact the sampled volume, but according to our estimates, it will not be enough to transform the estimated 0.33–18 mL into the previously suggested 1–3 L of screened blood volume. Since the CC was very well tolerated in our clinical study, increasing the time in circulation and increasing the surface area by changing the wire geometry may help to improve the performance of this novel device. However, the estimated blood volume screened with the CC was so far from the 2–2.5 L that can be screened with DLA and that parity between the two techniques does not seem attainable. A potential significant improvement of the method, more effectively exploiting the large blood volume passing the wire, could be achieved by adding *in vivo* immuno‐magnetic capturing, as recently suggested by Vermesh and colleagues (Ophir Vermesh *et al*., 2018).

The validity of CS as a prognostic marker has been widely reported for metastatic CRC. While our results corroborate the prognostic significance of CS‐positive status in M1 CRC patents, the detection of CTCs using CC in both M0 and M1 subgroups did not show any correlation to prognosis. Given the equal numbers of CTC detected in M0 and M1 patients using the CC, as well as the equal absolute number of detected CTCs between CS and CC, this result further indicates that the CTC detected using the CC might be of lower clinical interest.

The observed lack of correlation of CTC counts between CS and CC appears counterintuitive since both methods rely on EpCAM enrichment and use quite similar CTC detection methods. On the other hand, this was not completely unexpected, as similar observations were made in previous investigations of the CC in lung and prostate cancer (Gorges *et al*., 2016, Kuske *et al*., 2016). This lack of correlation can be in part explained by the uncontrollable endoluminal positioning of the CC wire, the interindividual differences in blood circulation, and the low CTC numbers and relatively small blood volume tested. Additionally, we cannot exclude that other, noncancerous cells (i.e., normal epithelial cells) might adhere to the CC, which then might be counted as CTCs. Another aspect concerns the delicate and somewhat subjective CTC identification on the CC wire. Although performed by trained personnel at GILUPI, microscopic observation of the rotating opaque wire appears difficult to standardize and likely leads to read‐out failures. In this context, it is also important to stress a potential weakness of our study, which is the lack of healthy controls. Healthy controls were not considered when our study was planned because all available data showed no false positives when the CC was applied *in vivo* in healthy controls (Gasiorowski *et al*., 2017; He *et al*., 2017; Li *et al*., 2017; Saucedo‐Zeni *et al*., 2012; Zhang *et al*., 2017). However, we cannot exclude the possibility of false positives in the present study, which might affect the prognostic impact of the detected cells.

In contrast, CS data evaluation is standardized, well documented, can be independently validated, and even objectified by software to reduce operator‐related variability (Swennenhuis *et al*., 2016). To improve the CC technology and to enable its routine clinical use, a fully automated detection appears mandatory. The observed divergence in CTC detection can also explain the missing correlation of CC CTCs with clinical parameters and prognostic information. We are confident that our CRC cohort was informative for testing the CC because CTCs detected in parallel by CS were significantly associated with advanced disease and poor outcome, as expected from previous reports (Cohen *et al*., 2008, 2009; Krebs *et al*., 2015; Romiti *et al*., 2014; Sastre *et al*., 2012). In this respect, our data are conflicting with a previous report by Theil and colleagues, reporting prognostic significance of CC CTCs in prostate cancer (Theil *et al*., 2016). A major reason might be that this group did not apply the wire *in vivo*. Instead, they used a 15 mL blood sample drawn from prostate cancer patients in a flow chamber with a defined constant diameter and a controlled flow of 1.2 cm·s^−1^ and applied the CC wire for 30 min to this flow device (Theil *et al*., 2016).

Clearly, the CC wire is able to capture cancer cells from the bloodstream, as verified by PCR‐detected tumor‐specific transcripts or mutated cancer genes (Gorges *et al*., 2016; Markou *et al*., 2018; Theil *et al*., 2016). Notably, these analyses were performed in bulk and did not allow to validate the identity of each single enumerated CTC. Nevertheless, such cancer‐specific molecular analyses should help to improve specificity of the current CC assay.

## 5 Conclusions

Collectively, our results suggest no outperformance of CS by the CC wire for detection of CRC CTCs and no biophysical fundament for the suggested high volume screened with CC. Nevertheless, an advantage of this innovative *in vivo* approach may be the possibility to establish a safe, easy‐to‐use, and rapid point‐of‐care CTC platform, if combined with molecular detection by quantitative RT‐PCR or digital PCR, which, according to previous reports, appear to be more suitable detection methods for the CC than the standard immunofluorescence staining used here.

## Conflict of interest

GILUPI GmbH, Potsdam, Germany, supplied the materials and performed blinded CTC identification on the CC. The authors declare no conflict of interest.

## Author contributions

LD, GF, RPN, TF, WTK, and NHS conceived and designed the study. LD, GF, GD, AR, and AK collected the samples and clinical data. EH, DN, and HN performed the analysis. LD, GF, GD, and RPN collected the raw data. LD, GF, GD, RPN, and NHS analyzed and interpreted the data. GF, NHS, LD, and GD drafted the manuscript. EH, RPN, AR, AK, HN, DN, TF, and WTK critically reviewed the manuscript for intellectual content. All authors reviewed and approved the final manuscript.

## Supporting information


**Appendix S1.** Supplemental Calculations**.**
**Fig. S1.** 3D model of vessel and wire with flow lines and slices showing fluid velocity.
**Fig. S2.** 3D model as in Figure S1, including slices used to calculate the flux past the Gilupi wire.

**Table S1.** Spearman's correlation of the CS and CC results of all 80 patients.
**Table S2.** Correlation of CTC detection with CS or CC using different cut‐offs and clinicopathological characteristics.
