# Supplementary material for: Detection of circulating tumor cells in colorectal cancer patients using the GILUPI CellCollector: results from a prospective, single‐center study
Source: Mol Oncol. 2019 Jun 17;13(7):1548–58. doi: 10.1002/1878-0261.12507 (PMC6599844; doi:10.1002/1878-0261.12507)
Supplement: Supplementary file 1 — Appendix S1. Supplemental Calculations. Fig. S1. 3D model of vessel and wire with flow lines and slices showing fluid velocity. Fig. S2. 3D model as in Figure S1, including slices used to calculate the flux past the Gilupi wire. [file MOL2-13-1548-s001.pdf]

Supplemental Calculations

Two types of in-silico models where used to approximate the volume passing the Gulipi wire in 30 minutes in vivo. We used the following conditions and assumptions for both models:

1. An average blood flow velocity ranging from 4 cm/s to 6 cm/s [1] and the cubital vein diameter ranging from 1.7 to 2.7 mm in diameter [2] before the insertion of the wire. We neglect any influence by the wire on these parameters.
2. Not all volume passing through the vein will be effectively sampled by the Gulipi wire. We choose an effective capture distance of 50 µm. As this distance is rather arbitrary and there is a lack of experimental data for this parameter, we also provide the calculated volumes for 25 and 75 µm capture distance in the results table below.

(1) The first model is a simple 2D theoretical model. Here, the flow past the Gilupi wire was modeled by Hagen Poiseuille flow through an annular section with no consideration for the shear thinning behavior of blood. The inner cylinder represents the Gilupi wire in the middle of the outer cylinder, the blood vessel wall. The volume passing the area that can effectively capture CTC can then be calculated by integrating the velocity profile described by the Hagen-Poiseuille flow [3]. This resulted in the following calculation:

We define the following constants

G	= 0.262 * 10^-3 (m)	radius of the Gilupi wire
R	= 1.1 * 10^-3 (m)	radius of the blood vessel
x	= 50 * 10^-6 (m)	capture distance of the Gilupi wire
µ	= 3.0 * 10^-3 (Pa · S)	dynamic viscosity
Q	= 0.050 *π*R^2 (m^3/s)	volume flow through the vein

Which we use to calculate the following values

k	=	G/R	ratio between the two diameters
dPdz	=	(Pa/m)	pressure drop/distance
v(r)	=	(m/s)	flow speed along z axis
vol(r)	=	(m^3/s)	volumetric flow rate across the CTC capturing distance
q	=	(ml /30 min)	volume passing the capturing distance per 30 minutes in ml

$$\mathrm{dPdz} = \frac{Q\,8\,\mu}{\pi R^4\left(1 - k^4 - \frac{(1-k^2)^2}{\ln\left(\frac{1}{k}\right)}\right)}$$

$$v(r) = \frac{\mathrm{dPdz}}{4\mu} R^2\left(1 - \left(\frac{r}{R}\right)^2 + \frac{1-k^2}{\ln\left(\frac{1}{k}\right)}\ln\left(\frac{r}{R}\right)\right)$$

$$\mathrm{vol}(r) = \int_G^{G+x} \frac{\mathrm{dPdz}}{4\mu} R^2\left(1 - \left(\frac{r}{R}\right)^2 + \frac{1-k^2}{\ln\left(\frac{1}{k}\right)}\ln\left(\frac{r}{R}\right)\right) 2\pi r\, dr$$

$$q = vol(r) \cdot 60 \cdot 30 \cdot 10^6$$

(2) The second model is a 3D finite element model solved using Comsol v3.6 (Comsol Multiphysics GmbH, Germany). The cubital vein was modeled as a cylinder and the Gulipi wire was inserted 1cm at an angle of 10 degrees. The flow at the inlet was assumed to be a fully developed laminar flow profile. the outlet as having no pressure. The flow was calculated using the solver for Navier-Stokes equations in the laminar regime. The dynamic viscosity of blood was modeled to be shear dependent as described by G. Mach et. al. [4]

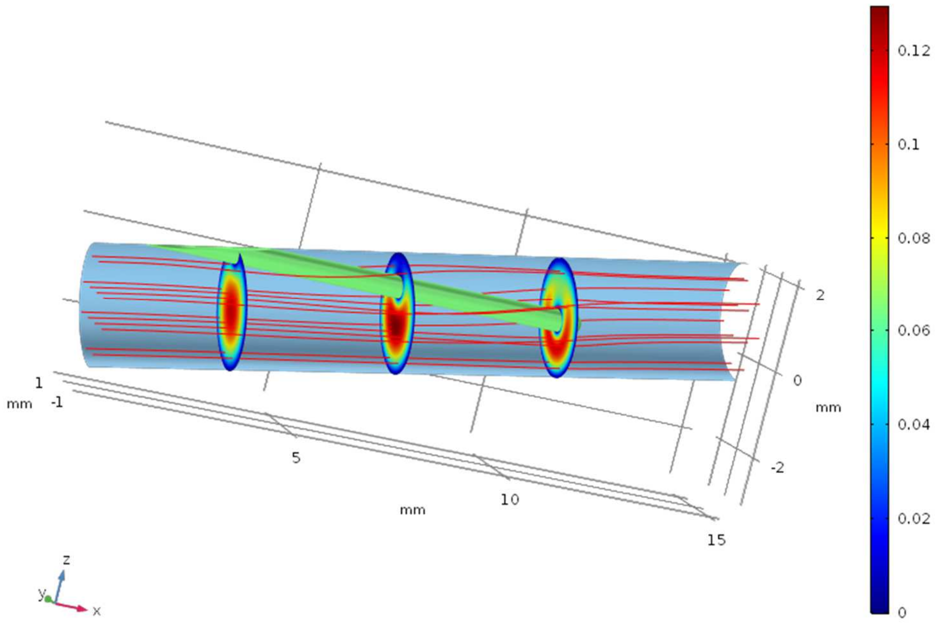

Figure S1: 3D model of vessel (blue) and wire (green) with flow lines (red) and slices showing fluid velocity (see scale on right). Calculated using Comsol v3.6.

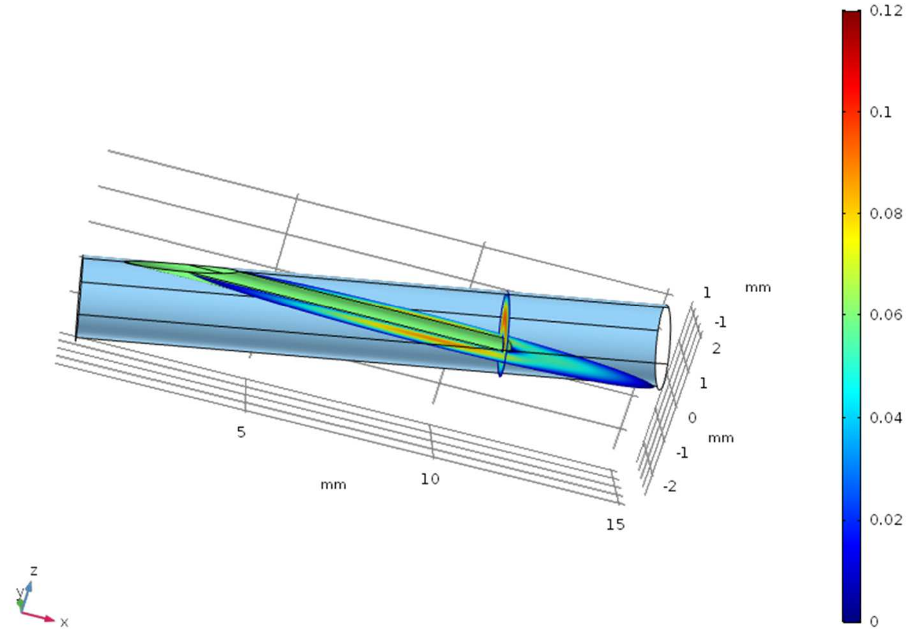

Figure S2: 3D model as in Figure S1, including slices used to calculate the flux past the Gilupi wire. Calculated using Comsol v3.6.

The volume sampled by the needle was calculated by integrating the flux through the planes shown in **Figure S2** using the noted capture distance from the wire.

It is important to note, when comparing these two models using the same capture distance, that the length of wire presented to the volume flowing past is different. This means that although the volume sampled is increased in the 3D model, the time the cells spend within the capture distance is shorter.

**Vein diameter**	**Average speed**	**Total blood volume in vein in 30 minutes**	**Sampled volume (ml) per 30 minutes at capture distance (µm)**					
(mm)	(cm/s)	(ml)	2D model			3D model		
			25µm	50µm	75µm	25µm	50µm	75µm
1.7	6	222	0,76	3,1	6,9	2,1	8,4	18
2.2	5	323	0,49	2,0	4,5	1,7	6,7	14
2.7	4	398	0,33	1,3	3,1	1,1	4,1	9,0
2.7	6	595	0,49	2,0	4,6	1,7	6,3	13,5

[1] OpenStax College. Anatomy and physiology. Chapter 20.2 Rice University, 2013.
[2] E. Korten, S. Spronk, M.T. Hoedt, G.M. de Jong, R.P. Tutein Nolthenius, Eur J Vasc Endovasc Surg 2009 Sep,38(3):375-80. doi: 10.1016/j.ejvs.2009.03.029. Epub 2009 May 21
[3] T. Papanastasiou, G. Georgiou, A. Alexandrou, Viscous Fluid Flow, CRC Press, Boca Raton, 1999.
[4] Mach, G., et al. "A Non Newtonian Model for Blood Flow behind a Flow Diverting Stent." Excerpt from the Proceedings of the
