# Supplementary material for: Detection of circulating tumor cells in colorectal cancer patients using the GILUPI CellCollector: results from a prospective, single‐center study
Source: Mol Oncol. 2019 Jun 17;13(7):1548–58. doi: 10.1002/1878-0261.12507 (PMC6599844; doi:10.1002/1878-0261.12507)
Supplement: Supplementary file 2 — Table S1. Spearman's correlation of the CS and CC results of all 80 patients. Table S2. Correlation of CTC detection with CS or CC using different cut‐offs and clinicopathological characteristics. [file MOL2-13-1548-s002.pdf]

Supplemental Tables:

Suppl Table 1: Spearman's correlation of CellCollector (CC) and CellSearch (CS) results

Variable	Patients	Correlation coefficient	p-value
CTC count (CC and CS)	80	-0.086	0.449
CTC positive (≥ 1 CTC) or negative (no CTC)	80	-0.017	0.880
CTC positive (≥ 2 CTC) or negative (<2 CTC)	80	-0.147	0.194
CTC positive (≥ 3 CTC) or negative (<3 CTC	80	-0.195	0.082

Table S1: Spearman's correlation of the CS and CC results of all 80 patients.

Suppl Table 2: Correlation between the prevalence of circulating tumor cells and clinicopathological parameters in colorectal cancer patients

	Cell Serach									Cell Collector								
**Patient subset**	**≥ 1 CTC**		p-value	**≥ 2 CTCs**		p-value	**≥ 3CTCs**		p-value	**≥ 1 CTC**		p-value	**≥ 2 CTCs**		p-value	**≥ 3CTCs**		p-value
**Age (yrs)**	**n**	**(%)**		**n**	**(%)**		**n**	**(%)**		**n**	**(%)**		**n**	**(%)**		**n**	**(%)**	
< 70 (n=38)	13	(34.2)	0.587	8	(21.1)	0.823	7	(18.4)	0.617	12	(32.6)	0.095	10	(26.3)	0.261	7	(18.4)	0.084
≥ 70 (n=42)	12	(28.6)		8	(19.0)		6	(14.3)		21	(50.0)		16	(38.1)		15	(35.7)	
**Sex**																		
Male (n=49)	18	(36.7)	0.183	12	(24.5)	0.207	9	(18.4)	0.519	21	(42.9)	0.714	16	(32.7)	0.971	13	(26.5)	0.807
Female (n=31)	7	(22.6)		4	(12.9)		4	(12.9)		12	(38.7)		10	(32.3)		9	(29.0)	
**Primary tumor**																		
Location																		
Colon (n=37)	15	(40.5)	0.212	12	(32.4)	0.077	9	(24.3)	0.256	14	(37.8)	0.765	12	(32.4)	0.788	9	(24.3)	0.952
Rectum (n=24)	6	(25.0)		3	(12.5)		3	(12.5)		10	(41.7)		7	(29.2)		6	(25.0)	
Tumor stage																		
T1/T2 (n=11)	1	(9.1)	0.051	1	(9.1)	0.187	0	(0.0)	0.070	5	(45.5)	0.647	4	(36.4)	0.680	3	(27.3)	0.819
T3/T4 (n=50)	20	(40.0)		14	(28.0)		12	(24.0)		19	(38.0)		15	(30.0)		12	(24.0)	
Lymph node																		
N0 (n=22)	2	(9.1)	**0.002**	1	(4.5)	**0.006**	0	(0.0)	**0.004**	8	(36.4)	0.720	6	(27.3)	0.624	5	(22.7)	0.800
N+ (n=39)	19	(48.7)		14	(35.9)		12	(30.8)		16	(41.0)		13	(33.3)		10	(25.6)	
Distant metastasis																		
M0 (n=29)	2	(6.9)	**< 0.001**	1	(3.4)	**< 0.001**	1	(3.4)	**0.002**	13	(44.8)	0.404	11	(37.9)	0.276	9	(31.0)	0.266
M+ (n=32)	19	(59.4)		14	(43.8)		11	(34.4)		11	(34.4)		8	(25.0)		6	(18.8)	
UICC stage																		
UICC I/II (n=17)	0	(0.0)	**< 0.001**	0	(0.0)	**0.006**	0	(0.0)	**0.016**	6	(35.3)	0.687	5	(29.4)	0.856	4	(23.5)	0.905
UICC III/IV (n=44)	21	(47.7)		15	(34.1)		12	(27.3)		18	(40.9)		14	(31.8)		11	(25.0)	
**Recurrent disease**																		
Local recurrence (n=2)	0	(0.0)	0.440	0	(0.0)	0.725	0	(0.0)	0.725	0	(0.0)	0.156	0	(0.0)	0.253	0	(0.0)	0.253
Distant recurrence (n=17)	4	(23.5)		1	(5.9)		1	(5.9)		9	(52.9)		7	(41.2)		7	(41.2)	
**Resection status**																		
R0 (n=60)	12	(20.0)	**< 0.001**	7	(11.7)	**0.001**	5	(8.3)	**0.001**	26	(43.3)	0.512	20	(33.3)	0.783	17	(28.3)	0.772
R1+R2 (n=20)	13	(65.0)		9	(45.0)		8	(40.0)		7	(35.0)		6	(30.0)		5	(25.0)	

Table S2: Correlation of CTC detection with CS or CC using different cut-offs and clinicopathological characteristics. P-values were calculated by Mann-Whitney test for numerical and the chi-square test for categorical data
